# The Battle between Virus and Host: Modulation of Toll-Like Receptor Signaling Pathways by Virus Infection

**DOI:** 10.1155/2010/184328

**Published:** 2010-06-16

**Authors:** Shin-ichi Yokota, Tamaki Okabayashi, Nobuhiro Fujii

**Affiliations:** Department of Microbiology, Sapporo Medical University School of Medicine, South-1, West-17, Chuo-ku, Sapporo 060-8556, Japan

## Abstract

In order to establish an infection, viruses need to either suppress or escape from host immune defense systems. Recent immunological research has focused on innate immunity as the first line of host defense, especially pattern recognition molecules such as Toll-like receptors (TLRs), RIG-I-like receptors (RLRs), and NOD-like receptors (NLRs). Various microbial components are recognized by their vague and common molecular shapes so-called, pathogen-associated molecular patterns (PAMPs). PAMPs induce inflammatory reactions mediated by the activation of the transcription factor, NF-*κ*B, and by interferons, which lead to an antiviral immune response. Viruses have the capacity to suppress or escape from this pattern recognition molecule-mediated antimicrobial response in various ways. In this paper, we review the various strategies used by viruses to modulate the pattern recognition molecule-mediated innate immune response.

## 1. Introduction

The host immune system recognizes and eliminates invading pathogenic microorganisms such as viruses, bacteria, and fungi. The first line of defense in mammals is the innate immune system. Recently, the mechanisms by which the innate immune system recognizes pathogen have been extensively studied. Pattern recognition molecules/pathogen recognition receptors (PRRs) are classified into three families: Toll-like receptors (TLRs), RIG-I-like receptors (RLRs), and nucleotide binding-oligomerization domain (NOD)-like receptors (NLRs) [1, 2]. Ten TLRs (TLR1 to 10) have been identified in humans. The RLR family contains retinoic acid-inducible gene I (RIG-I) and melanoma differentiation associated gene 5 (MDA5) [3]. The NLR receptor family contains NOD1, NOD2, NLRP3, NLRPC5, NLRP1, NAIP, and CIITA [4]. In addition, DNA-dependent activator of interferon regulatory factors (DAI) has been identified as a DNA sensor [5]. Various microbial components are recognized as their vague and common molecular shapes by PRRs. Early responses against virus infection are initiated on recognition of pathogen-associated molecular patterns (PAMPs) by pattern recognition molecules, triggering two responses. One is the production of interferons (IFNs) resulting in an antiviral state as part of the innate immune response, and the second is maturation of dendritic cells (DCs) to establish acquired immunity. In order to establish an infection within a host, viruses must escape from and/or suppress the immune system by various strategies. An important strategy used by viruses is modulation of PAMP-induced immune responses.

TLR signaling proceeds via two pathways: the myeloid differentiation factor 88 (MyD88)-mediated pathway, and the Toll-interleukin-1 receptor (TIR)-domain-containing adaptor inducing IFN-*β* (TRIF)-mediated pathway [1, 2]. The former causes activation of the transcription factor NF-*κ*B, which activates various genes contributing inflammatory reactions. The latter causes induction of IFNs, whose stimulation leads cells to antiviral state. TLR3 only activates the TRIF-mediated pathway. TLR3 signaling activates IRF-3, an important transcription factor for IFN-*β*, and IFN production is induced. TLR2 only activates the MyD88-mediated pathway. However, TLR4 activates both pathways, so TLR4 agonists activate NF-*κ*B and induce IFN production. Cytosolic PRRs, such as RIG-I, MDA5, and DAI, commonly activate IRF-3. The expression of PRRs differs depending on the cell type. Importantly, it is different between cells derived from myeloid stem cells (myeloid dendritic cells (mDCs), monocytes, macrophages, Langerhans cells, and neutrophils) and cells derived from lymphatic stem cells (plasmacytoid dendritic cells (pDCs), T cells, and B cells). For example, TLR7 and TLR9 are rarely expressed on mDCs, whereas TLR3 and TLR8 are rarely expressed on pDCs. TLR4, on the other hand, is expressed at very low levels on both pDCs and mDCs.

## 2. Viruses and Innate Immunity

**Figure 1:**
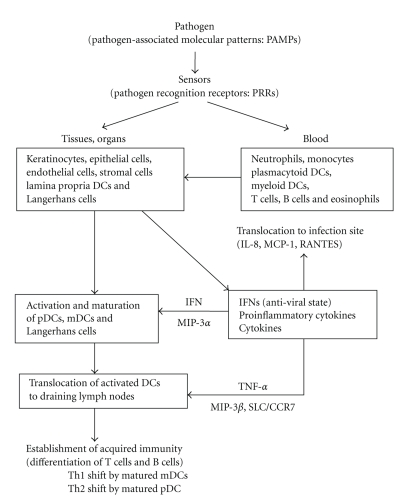
Process of innate immunity to acquired immunity.

Initially, viruses invade the host epithelial tissues found in the oral cavity, respiratory tract, intestinal tract, and the urogenital apparatus (Figure 1). Lamina propria DCs, Langerhans cells, and stromal cells are resident in these tissues. In the connective tissues, fibroblasts resident, and capillary vessel and lymphatic vessel are expanded. Monocytes, macrophages, T cells, B cells, pDC, and mDC circulate within the blood vessels and lymphatic vessels, and patrol the interstitial spaces. All these cells are potential targets for virus infection. Virus-infected epithelial cells and fibroblasts produce IFNs, mainly IFN-*β* and IFN-*λ*, which provide surrounding uninfected cells with antiviral state. Furthermore, chemokines and cytokines, such as interleukin-1*β* (IL-1*β*), IL-6, IL-8, granulocyte-macrophage colony-stimulating factor (GM-CSF), and tumor necrosis factor-*α* (TNF-*α*) are also produced. These molecules promote chemotaxis of the resident DCs (lamina propria DCs and Langerhans cells) toward virus-infected and dead cells. Neutrophils, monocytes, macrophages, plasma cells, mDCs, and pDCs also migrate from the blood vessels to the site of infection. IFN-*γ*-inducible protein 10 (IP-10), monocyte chemotactic protein 1 (MCP-1), macrophage inflammatory protein-2 (MIP-2), MIP-3*α*, and MIP-3*β* largely contribute to the transmigration of blood DCs. However, these blood-borne immune cells can also be infected by viruses, which can then modulate the production of various cytokines and chemokines. 

To establish an infection, viruses need to suppress a number of host immune responses, the antiviral activity induced by IFNs, the chemotaxis of immune cells induced by chemokines/cytokines, the maturation and activation of DCs, activation of NK and NKT cells, transmigration of mature DCs to the lymph nodes, and the differentiation and activation of T cells and B cells in the lymph nodes, for example. When viruses infect immune cells, such as DCs, the infected cells frequently show suppression of maturation and differentiation, suppression of cytokine receptor and costimulatory molecule expression, secretion of molecules that mimic cytokines and cytokine receptors, and so on. Furthermore, infected cells often alter their cytokine profiles. These strategies are used by the virus to inhibit the acquired immune response. In addition, it has been suggested that virus infection induces regulatory T cells.

## 3. RNA Virus

### 3.1. Human T Lymphotropic Virus Type 1 (HTLV-1)

HTLV-1, which is a retrovirus, infects CD4^+^ T lymphocytes, CD8^+^ T lymphocytes, DCs, B cells, macrophages, and astrocytes, and preferentially replicates in CD4^+^ T lymphocytes. HTLV-1 causes latent infection as a provirus, whose genome is integrated into the host DNA, and does not replicate in cells in G0 phase. When the infected T lymphocytes are stimulated with antigen presentation from DCs, they proliferate triggering HTLV-1 replication. During the replication stage, a viral protein, Tax, activates NF-*κ*B and promotes the growth of infected cells via upregulation of IL-2 and IL-2 receptors [6, 7]. NF-*κ*B also activates the long terminal repeat (LTR) of HTLV-1 genome, which further enhances viral replication [8]. The replicated virus induces a host immune response, and cells infected with virus are eliminated by the induction of cytotoxic T cells specific for HTLV-1 Tax. In patients with HTLV-1-associated myelopathy/tropical spastic paraparesis (HAM/TSP), the high proviral load induces a strong HTLV-1-specific immune response [9]. This leads to the rapid elimination of infected cells through the induction of proinflammatory cytokines and cytotoxic T lymphocytes. So, in order to escape from acquired immune responses, HTLV-1 needs minimum replication and latent infection.

Some virus proteins are known as negative regulators of replication. The HTLV-1 basic leucine-zipper factor (HBZ) protein suppresses Tax-mediated transcription activation of the viral LTR [10]. The p30 protein suppresses transcription of mRNAs encoding Tax and Rev [11]. p30 also contributes to the expression of TLR2, TLR4, and TLR9, and activation of IRF4. In monocytes, the TLR2 gene promoter is regulated by the transcription factors Sp1, Sp3, and PU.1 [12], and the TLR4 gene by ISRE and PU.1 [13]. p30 downregulates the expression of both TLR2 and TLR4, because it binds to PU.1 and prevents it from binding to DNA [14]. This leads to the suppression of DC maturation, and of their subsequent migration to the lymph nodes. Furthermore, p30 suppresses the enzymatic activity of glycogen synthetase kinase 3*β* (GSK3*β*) through promotion of the phosphorylation of nine serine residues. This leads to the induction of IL-10, which suppresses the function of macrophages, and also the maturation and activation of DCs. In fact, serum IL-10 levels are elevated in patients with adult T lymphocyte leukemia. These immunosuppressive properties of IL-10 indirectly contribute to the inhibition of virus replication and to the suppression of virus-induced immune responses. Activation of infected T lymphocytes by DCs in the lymph nodes and cell-to-cell transmission of virus are considered to be important for virus proliferation in human. The viral p12 protein suppresses cell surface expression of both MHC class I and the IL-2 receptor, and also suppresses linker for activation of T cells (LAT), which is an adaptor protein required for T cell activation [15]. This results in suppression of T cells and dystunction of the stimulation/activation by DCs via the T cell receptor. HTLV-1 causes proliferation of infected cells rather than virus and suppression of host immune responses, which helps it to maintain a latent infection in order to survive.

### 3.2. Human Immunodeficiency Virus (HIV)

HIV is another retrovirus that uses similar escape strategies to HTLV-1. HIV can infect CD4^+^ T lymphocytes, monocytes, macrophages, and DCs, and preferentially replicates in activated T lymphocytes and activated macrophages. It replicates generally much more slowly in DCs regardless of maturation stage. HIV establishes a latent infection in resting T lymphocyte, and T lymphocyte activation by DCs or stimulation with IL-2 is thought to trigger active replication of the virus. Resting T lymphocytes show resistance to infection and proliferation of HIV. Apolipoprotein B mRNA editing enzyme catalytic polypeptide-like 3G (APOBEC3G) is important for this resistance [16, 17]. The antiviral mechanisms of APBEC3G are considered to act via inhibition of viral reverse transcriptase (RT). It also produces a transition (G to A) in the DNA strand transcribed by RT due to its cytidine deaminase activity. However, a mutant lacking cytidine deaminase retains antiviral activity. Low molecular weight complexes of APOBEC3G (LMM: 70–100 kDa) have antiviral activity, but high molecular weight complexes (HMM: 700 kDa) do not. The Vif protein of HIV converts LMM to HMM, and promotes proteasome-dependent degradation of the complex, so HIV can counteract its antiviral activity [18]. T cell activation leads to conversion of LMM to HMM (proviral environment). On the other hand, IFNs induce LMM type (antiviral environment) [19, 20]. 

 During the early stage of HIV infection, clinical symptoms show sings of immune system activation such as flu-like symptoms, rather than immunosuppression. Immunological activation is caused by RNA40, an oligonucleotide derived from HIV, which activates pDCs, mDCs, T lymphocytes, and monocytes via TLR7 and TLR8 [21]. The virus proteins, Tat and Vpr, induce proinflammatory cytokines via activation of NF-*κ*B [22, 23], which then enhances the transcription of the virus genome via the LTR. IL-8 also contributes to the propagation of the virus via the accumulation of T lymphocytes. 

Enhanced proliferation of HIV causes immune responses that attempt to eliminate the virus. So, HIV maintains a latent infection in order to survive within the host. The Vpu protein suppresses NF-*κ*B activation by inhibition of proteasome-dependent degradation of I*κ*B [24]. The Nef protein suppresses phosphorylation of ERK through induction of a phosphatase MKP-1 [25]. This causes suppression of TNF-*α* production by macrophages via TLR4 signaling. Because TNF-*α* is necessary for maturation and translocation of DCs (including Langerhans cells) to the lymph nodes, downregulation of TNF-*α* leads to suppression of acquired immune responses, and so prevents inhibition of virus proliferation by activated T lymphoctes. Furthermore, Nef inhibits T cell receptor induced lymphocyte activation [26]. These strategies contribute to the suppression of HIV proliferation in the lymph nodes, and also inhibit the propagation of infection. Other immunosuppressive mechanisms used by HIV have also been reported. Cosuppressive molecules, such as B7-H1 on DCs and PD1 on T lymphocytes, are upregulated in patients with HIV, and induce apoptosis through their interaction with DCs and T lymphocytes. In addition, HIV is thought to induce regulatory T cells [27].

### 3.3. Hepatitis C Virus (HCV)

HCV does not only infect hepatocytes. It can also infect DCs, macrophages, monocytes, and T lymphocytes. However, the virus does not replicate efficiently in these cell types. Although, virus particles and virus proteins are found in the blood of HCV patients. HCV causes no cytopathic effects. Immune responses against HCV may be weak, but infected cells are attacked and eliminated. In order to survive in the host, HCV maintains a chronic infection by suppressing the host immune responses. Both HCV core and NS3 proteins activate NF-*κ*B and AP-1 via stimulation of TLR2, which requires TLR1 and TLR6 as costimulators, in monocytes and Kupffer cells [28]. This activation leads to the production of IL-10 and TNF-*α*, both found in HCV patients at a high titer. IL-10 suppresses the maturation of pDCs and the activation of T lymphocytes, and induces apoptosis in pDCs. The NS3-NS4A protein complex, which is a serine protease, degrades TRIF and IPS-1/Cardif/MAVS/VISA, which are essential for cellular signaling via TLR3, TLR4, and RIG-I [29]. This shutting off of TRIF- and IPS-1-dependent signaling results in the suppression of IFN-*α* and IFN-*β* production, and in dysfunction of mDCs.

 The NS5A protein suppresses activation of IRAK-1 through its interaction with MyD88. This leads to the shutting off of TLR7 and TLR8 signaling, and to the suppression of maturation and differentiation of pDCs [30]. On the other hand, NF5A suppresses TRAF2 dependent NF-*κ*B activation via interaction with NF5A and TRAF2, but suppress neither MEK1 activation nor IKK*β*-dependent NF-*κ*B activation [31]. TNF-*α*-dependent activation of JNK is enhanced. Infected DCs are thought to affect the production of TNF-*α*, TNF-*α* signal transduction, and chemotaxsis and maturation. Dysfunction of DCs, suppression of T lymphocyte activation, and a decrease in DC number due to apoptosis allow HCV to establish a chronic infection [32]. It has also been reported that HCV-specific cytotoxic T lymphocytes share upregulated expression of the coinhibitory molecule PD-1, and that signaling from PD-1L (PD-1 ligand) on DCs results in suppression of HCV-specific cytotoxic T lymphocytes [33].

### 3.4. Measles Virus

Measles virus infection causes strong immunosuppression. Measles virus wild strains (clinical isolates) recognize CD150/SLAM (signaling lymphocyte activation molecule) as a receptor. However, laboratory (vaccine) strains only recognize CD46. SLAM is strongly expresseed on memory T cells and B cells, but is also expressed on monocytes, T cells, B cells, and matured DCs. SLAM is not expressed on immature DCs, epithelial cells, and endothelial cells. On the other hand, CD46 is expressed on variety of cell types. Infection of SLAM-negative mucosal epithelial cells with measles virus wild strains is thought to be mediated by an as yet unknown “third” receptor [34, 35]. The HA protein of wild virus strain, but not the laboratory strain, induces cytokines such as IL-1*α*, IL-1*β*, IL-6, IL-8, and IL-12 via the TLR2 signaling pathway [36]. These cytokines activate and recruit immune cells to the site of inflammation, where the activated immune cells are infected with measles virus via SLAM. The infection and cytokine production are thus propagated. 

 Interaction of the HA protein with SLAM suppresses TLR4-mediated IL-12 production, but not IL-6 and TNF-*α* production. However, IL-12 production mediated by other TLR signaling pathways (i.e., not via TLR4) is unaffected by the HA protein [37]. These observations suggest that SLAM is a coupling factor for TLR4, and that the HA protein inhibits this function. The HA protein also interacts with a C-type lectin, dendritic cell-specific ICAM-3-grabbing nonintegrin (DC-SIGN) on DCs, and activates the serine/threonine protein kinase Raf-1 via the Ras signaling pathway. DC-SIGN-mediated Raf-1 activation induces phosphorylation of NF-*κ*Bp65 on Ser-276, and its subsequent acetylation. This leads to the enhanced transcription of IL-10 [38]. During infection stage that virus proteins are synthesized de novo, the host cells, such as monocytes and DCs, infected with measles virus show suppressed IL-12 production and TLR signaling, via TLR2, TLR4, TLR7, and TLR9. pDCs infected with the measles virus showed suppressed IFN production and dysfunction of maturation [39, 40]. 

Both monocytic cell lines U937 and THP-1 and human peripheral blood mononuclear cells infected with measles virus show markedly suppressed TLR2- and TLR4-mediated proinflammatory cytokine induction via NF-*κ*B and AP-1 [41]. However, epithelial cells infected with measles virus show constitutive activation of NF-*κ*B and proinflammatory cytokine production, and these are further enhanced by treatment with TLR agonists such as lipopolysaccharide (LPS). Monocytic cell lines infected with the mumps virus, which also belongs to the Orthomyxoviridae family, show constitutive activation of NF-*κ*B and constitutively high levels of IL-8 production. In monocytic cells infected with the measles virus, LPS-induced ubiquitination (probably K63-linked type) of TNF receptor-associated factor 6 (TRAF6) is suppressed and does not form active complexes of TAK1, TAB2, and TRAF6. An ubiquitin-modifying enzyme A20, which is a host NF-*κ*B negative regulator, is upregulated in measles virus-infected monocytic cells, but not in infected epithelial cells. The promoter region of the A20 gene shares two NF-*κ*B binding sites and a negative regulatory motif, ELIE, which is located upstream of, and adjacent to the two NF-*κ*B binding motifs. Measles virus P protein (phosphoprotein) interacts with the ELIE motif, and activates transcription of A20. P protein is thought to release the suppressed A20 transcription machinery independently of activated NF-*κ*B [42]. The reason for cell-type specific A20 expression is unclear. However, the measles virus V protein, which is formed by RNA editing of the P genome and has an N terminal amino acid sequence identical to that of P protein, does activate NF-*κ*B. The balance of the expression levels and time courses of the P and V proteins may also contribute to the cell-type specific suppression of TLR signaling pathways.

### 3.5. Influenza Virus

The NS1 protein of type A influenza viruses suppresses innate immune system signaling activated by the PAMPs, via TLR3, TLR4, RIG-I, and MDA5 system. The suppression should contribute to efficient replication of infected virus in respiratory epithelial. The mechanism of suppression is mainly via inhibition of IRF-3 phosphorylation [43], leading to suppressed induction of IFN-*α*/*β*, and IFN-*λ*1, 2, and 3. In addition, inhibition of NF-*κ*B and AP-1 activation in influenza virus infected cells leads to suppressed proinflammatory cytokine production, for example, IL-8 and TNF-*α*. Influenza virus NS1 protein is known to be a multifunctional protein able to inhibit the type I IFN induction, the IFN-induced antiviral activity, the binding and sequestration of dsRNA, the interference with the host mRNA processing, the facilitation of preferential viral mRNA translation, and the inhibition of DC activation [44, 45].

### 3.6. Human Respiratory Syncytial Virus (RSV)

RSV F protein causes TLR4-mediated NF-*κ*B activation during the early infection stages of infection that is dependent upon virus replication. IL-1*β*, IL-6, and IL-8 are induced via NF-*κ*B activated by the stimulation of TLR4. During the late stages, the viral G protein is produced, and secreted, which then suppresses TLR4-mediated signal transduction [46]. The cysteine-rich (GCRR) region of the G protein is also important for NF-*κ*B suppression. Following the interaction of virus proteins with host cell surface proteins, the viral M2-1 protein inhibits the translocation of RelA, a component of NF-*κ*B, to the cell nucleus [47]. Expression of the viral nonstructural proteins, NS1 and NS2 inhibit activation of IRF3 induced by TLR3, TLR4, and RIG-I signaling, and also suppress IFN production [48]. However, NS1 and NS2 proteins have little effect on NF-*κ*B or AP-1 activation, so the production of proinflammatory cytokines may be effectively induced in the RSV-infected cells.

## 4. DNA Virus

### 4.1. Vaccinia Virus

Orthopox viruses, including the vaccinia virus, produce proteins that mimic cytokine receptors, such as those for IFN-*α*, IFN-*β*, IFN-*γ*, IL-1*β*, IL-18, and TNF-*α*, and disturb the cytokine signal transduction system. Other viral proteins also disturb the intracellular signal transduction systems. The viral A46R protein has a TIR domain, and interacts with MyD88 and TRIF, suppressing both IL-1 and TLR signal transduction, but not TNF-*α* signal transduction [49]. The viral A52R protein binds to IRAK2, suppresses TRAF6-dependent IKK and NF-*κ*B activation, and then inhibits production of IL-8 and RANTES. However, A52R also binds to TRAF6 and promotes polyubiquitination of TRAF6, TAK1 activation, MAPKK6 phosphorylation, and activation of the JNK-p38 MAP kinase pathway. The later leads to the induction of IL-10 production [50]. The viral N1L protein interacts with the IKK complex (IKK*α*-IKK*β*-IKK*γ*), TBK1, and IKK*ε*, and then suppresses the activation of NF-*κ*B and IRF-3 [51]. Vaccinia virus not only suppresses proinflammatory cytokines, but also induces production of an immunosuppressive cytokine IL-10, which shifts the Th1 response and suppresses cellular immunity. Human monocytes infected with vaccinia virus produce IL-10, and this IL-10 is then further upregulated by stimulation with LPS [52].

### 4.2. Adenovirus

During the early stages of infection, adenovirus particles induce the MyD88-dependent production of RANTES, IP-10, and MIP-1 [53]. The cytokines produced enhance both sensitivity to LPS and the production of TNF-*α*. TNF-*α*  suppresses the formation and maturation of virus particles, and induces apoptosis of infected cells. TNF-*α* also promotes the chemotaxis and maturation of dendritic cells. The induction of TNF-*α* is considered to be a host defense response. On the other hand, the adenovirus E1A and E3 proteins inhibit TNF-*α*-induced apoptosis. The receptor internalization and degradation (RID) complex, which consists two E3 products, E3(10.4 k)/RID*α* and E3(14.5 k)/RID*β*, suppresses cell surface expression of Fas, TNF-related apoptosis-inducing ligand (TRAIL) receptor 1, TRAIL receptor 2, and TNF receptor 1 [54, 55]. This results in the shutting down of the TNF-*α*-mediated signaling pathways in the infected cells. RID also suppresses the TLR4 signaling pathway. LPS-induced MCP-1 and IL-8 production are suppressed through the inhibition of NF-*κ*B and AP-1 activation [56]. RID does not alter the expression levels of TLR4, and so is thought to affect other components of the TLR signal transduction pathway.

### 4.3. Human Cytomegalovirus (HCMV)

HCMV modulates NF-*κ*B activity during the various stages of infection. During the early stages, the membrane glycoproteins gB and gH interact with TLR2 and activate NF-*κ*B [57]. NF-*κ*B then contributes to the induction of proinflammatory cytokines, to the expression of virus immediate early genes, and to the replication of the viral genome. The viral US28 protein, which is a HCMV-encoded chemokine receptor, constitutively activates both NF-*κ*B and phospholipase C signaling pathways [58]. The activated NF-*κ*B mediates the upregulation of the host serine/threonine protein kinase, receptor-interacting protein-like interacting caspase-like apoptosis regulatory protein kinase (RICK). RICK, which has a caspase-recruitment (CARD) domain, functions downstream of the pattern recognition receptors (which include the TLR, RLR, and NLR family members) and mediates NF-*κ*B and MAP kinase activation. RICK suppresses the replication of HCMV in cooperation with active NF-*κ*B and IFN-*β* [59]. Not to be outdone, the late gene products of HCMV also suppress NF-*κ*B activation [60, 61]. TNF-*α* signaling is suppressed through downregulation of TNF receptor 1. IL-6, IL-8, and MCP are not induced in HCMV-infected cells and chemotaxsis, activation, and maturation are all suppressed.

### 4.4. Epstein-Baar Virus (EBV)

EBV mainly infects B cells, although it can also infect T cells, NK cells, and epithelial cells. Both the attachment of EBV to receptor CD21 and the interaction of the virus glycoprotein gp350-gp250 with TLR2 activate NF-*κ*B [62]. The virus gB and gH proteins are also candidates for TLR2 ligands. Immortalization of B cells by EBV infection is due to the activation of NF-*κ*B by the latent membrane protein 1 (LMP1), and antigen stimulation-like signaling of the B cell receptor by LMP-2. TLR2 signaling suppresses the transcription of the TLR9 gene via activation of NF-*κ*B containing p65 protein. So downregulation of TLR9 and upregulation of TLR7 and MyD88 are observed in EBV-infected cells [63]. Cell proliferation is thought to be driven by TLR7 signaling activated by virus RNA, because the TLR7 antagonist IRS661 suppresses cell division. Small RNA encoded by the EBV genome (EBV-EBERs) activates TLR7 signaling, and induces IL-10 production. TLR7 signaling also upregulates and activates IRF-5, and induces proinflammatory cytokines. However, activated IRF-5 is negatively regulated by EBV-induced IRF-4 and a splice variant of IRF-5 (V12IRF-5) [63]. This suggests that the TLR7 signaling system play a role in the cell division of EBV-infected cells, and in the establishment of persistent infection. Lytic infection and production of virus particles are observed during the late stage of EBV infection. Late proteins suppress NF-*κ*B activation. This leads to the downregulation of proinflammatory cytokines, the upregulation of TLR9, and the suppression of TLR7 function through interaction of TLR7 and TLR9. So the TLR9 system should be important during late stage of EBV infection.

## 5. Concluding Remarks

Viruses are in a constant battle with the host immune system. Viruses modulate both the innate and acquired immune systems using a number of clever strategies. The goal appears to be to survive within the host for a long time, rather than efficient replication. Excessive replication would lead to detection and elimination by the host innate and acquired immune systems, thus bringing about the death of the virus. The virus needs to strike a balance between activation and suppression of host immune response. It is likely that each virus has developed various strategies to modulate the host immune response individually, and viruses that have succeeded in creating a good balance between host and parasite have survived.

Modification of TLR signaling is a promising strategy for treatment of cancer, allergy, and infectious diseases [64, 65]. Especially, the immunomodulators should not generate resistant virus to drugs compared to antiviral drugs targeting viral proteins. As an existing example of virus infection, imiquimod, which is a TLR7 agonist, have been applied to an infectious disease caused by human papilloma virus, namely, condylomata acuminata [66]. TLR4 antagonists are trying to be applied to treatment of sepsis. Several issues must be considered for the clinical application of TLR signaling. Among the greatest is assessment of what are keys of host defense or virus survival in view of a series of viral infection process. For example, human herpes simplex virus 1 (HSV-1) requires activated NF-*κ*B for its efficient replication [67]. On the other hand, NF-*κ*B has a key role in inflammatory reactions via transcription activation of proinflammatory cytokines, cell adhesion molecules, and MHC. Thus, activation of NF-*κ*B is a double-edged sword for HSV-1. In the host side, TLR signaling varies according to organs and tissues. For example, intestinal epithelial cells express low levels of TLRs and high levels of negative regulators of TLR signaling, such as Tollip [68, 69]. Also, in intestinal immunity, NF-*κ*B activation in a subset TLR signaling in DCs and macrophages is suppressed by a negative regulation I*κ*BNS [70]. The dysregulation of TLR signaling in the lumen of intestinal epithelial causes limit chronic inflammatory activation induced by commensal bacteria. Various TLR polymorphisms have been found, and some of them are shown to contribute to dysregulation of TLR signaling. The dysregulation has been suggested to be linked with a number of disease sensitivity and condition depending on individual differences [71, 72].

In conclusion, the molecular mechanisms involved in modulation of host immune systems, including TLR signaling, give us important hints on how to overcome infectious diseases caused by viruses.

## Figures and Tables

**Figure 2:**
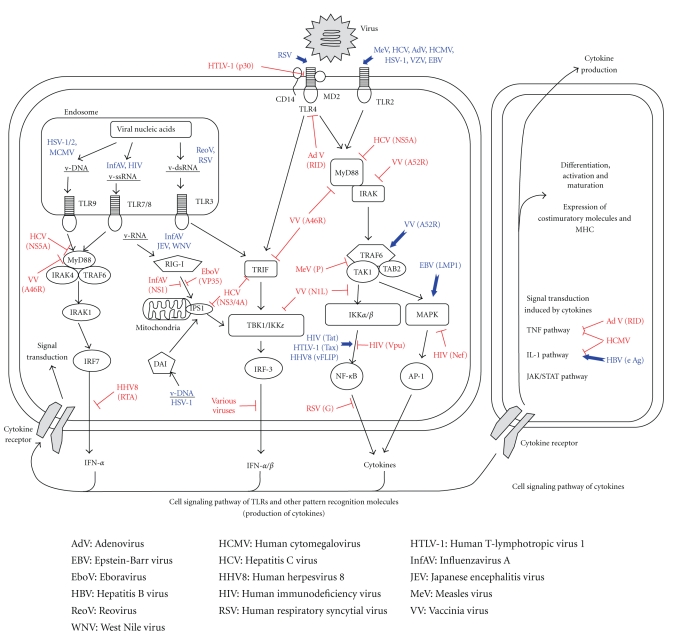
Modulation of TLR and other PAMP-induced signal transduction pathways by virus infection. Red line indicates suppression by virus. Blue line indicates activation by virus. Parenthesises denote viral proteins ornucleic acids.

**Table 1:** Molecular mechanisms involved in modulation of host innate immune systems by viruses.

Virus/Virus protein	Host pathway	Function	Effect
RNA virus			

Human immunodeficiency virus (HIV)			
Nef	TCR-CD30	Inhibit NF-*κ*B and AP-1 activation	Inhibit T cell activation
	TLR4	Inhibit ERK activation (Dephosphorylation of ERK by induced MKP-1)	Suppress TNF-*α* production
Vpu	TLRs	Inhibit NF-*κ*B activation (Stabilize I*κ*B)	Suppress cytokine production
Tat		Activate NF-*κ*B (PKR-dependent)	
Vpr		Activate NF-*κ*B	Enhance IL-6, IL-8, and IL-10 production
RNA40	TLR7/8	Activate IRF-7 mediated by MyD88	Activate pDCs

Human T-lymphotropic leukemia virus 1 (HTLV-1)			
p30	TLR4	Suppress TLR4 expression (Suppress PU.1 fuction)	Suppress cytokine production
		Inhibit GSK3-*β*	Enhance IL-10 production
p12	TCR	Suppress phosphorylation of PLC-*γ*1 and Vav	Suppress T cell activation by DCs
Tax		Activate IKK (Bind to IKK*γ*)	Induce IL-2, IL-2R, GM-CSF, and IL-15

Hepatitis C virus (HCV)			
NS3/4A	TLR3/4	Degrade TRIF	Suppress IFN-*α*/*β* production
			Suppress CD4^+^ T cell activation by dysfunction of mDCs
NS5A	TLRs	Inhibit NF-*κ*B activation (Bind to MyD88)	Suppress cytokine production
		Inhibit JNK activation (Bind to TRAF2)	Suppress TNF pathway activation
core, NS3	TLR2	Activate MAP kinase pathway, NF-*κ*B, and AP-1	Dysfunction of pDCs by enhanced production of IL-10 and TNF- *α*

Measles virus			
N (?)			Suppress IL-12 production mediated by TLR4 in DCs (Suppress Th1 differentiation)
P	TLR2/4	Suppress NF-*κ*B and AP-1 activation (Upregulate host NF-*κ*B negative regulator A20)	Inhibit proinflammatory cytokine and chemokine production
HA	TLR2	Activate NF-*κ*B	Induce cytokine production

Influenza virus A			
NS1	TLR3/7/9	Suppress of IRF3/7 activation	Inhibit IFN production in pDC
	TLR4	Suppress of NF-*κ*B and AP-1 activation	Suppress DC and T cell activation
	RIG-I	Suppress of IRF3/7 activation	Inhibit IFN production in mDC
SARS-coronavirus (SARS-CoV)			
N		Activate AP-1 Bind to NF-*κ*B and C/EBP binding motifs of IL-6 promoter	Induce IL-6 and COX-2

	promoter		

Human respiratory syncytial virus (RSV)			
G	TLR2/4/9	Suppress NF-*κ*B activation	Suppress cytokine production
F	TLR4	Activate NF-*κ*B	Induce IL-1, IL-6, IL-8, and RANTES
M2-1		Activate NF-*κ*B	
?	TLR7/9		Suppress IFN production in pDC
NS1, NS2	TLR3/4, RIG-I	Suppress IRF3 activation	Suppress IFN production in mDC

Rotavirus			
VP4		Activate NF-*κ*B Suppress JNK (Bind to TRAF2)	

DNA virus			

Vaccinia virus			
A46R	TLRs	Suppress NF-*κ*B activation (Bind to MyD88, TRAM, and TRIF)	Suppress cytokine production
A52R	TLRs	Suppress NF-*κ*B activation (Bind to IRAK2)	Suppress cytokine production
		Activate p38 MAPK and JNK (Bind to TRAF6)	Induce IL-10 production
N1L	TLRs	Suppress NF-*κ*B activation (Bind to IKK*γ*)	Suppress cytokine production
		Suppress IRF3 activation (Bind to TBK1)	Suppress IFN production

Adenovirus			
RID	TLR4	Suppress NF-*κ*B and AP-1 activation	Suppress MCP-1 and IL-8 production
	TNF pathway	Suppress TNFR1 expression	Suppress MCP-1 and IL-8 production
capsid		Activate NF-*κ*B, ERK, and MAPK	Induce RANTES and IL-10

Herpes simplex virus (HSV)			
virion	TLR2		Induce cytokine production
viral DNA	TLR9		Induce IFN production

Varicella-zoster virus			
vrion	TLR2		Induce cytokine production

Human cytomegalovirus (HCMV)			
late proteins	IL-1 system	Suppress NF-*κ*B activation (Inhibit site located upstream of MAP3K)	
	TNF system	Suppress TNFR1 expression	
gB/gH	TLR2/1	Activate NF-*κ*B	Produce proinflammatory cytokines
Epstein-Barr virus (EBV)			
late protein		Suppress NF-*κ*B activation	Inhibit COX-2 activation
gp350/gp220	TLR2/CD21	Activate NF-*κ*B	Induce MCP-1 production
LMP-1		Activate p38 MAPK (TRAF6-dependent)	Suppress DC function by induced IL-10

Human herpesvirus 8 (HHV8)			
vFLIP		Activate IKK	
		(Bind to IKK*γ*)	

RTA		Degrade IRF7 by proteasome system	Suppress IFN-*α*/*β* production

Hepatitis B virus			
e antigen	IL-1 pathway	Activate NF-*κ*B	Induce cytokine
		(Bind to mIL-1RAcP)	production
